# Case Report: Thrombotic and haemorrhagic disseminated intravascular coagulation in COVID-19

**DOI:** 10.3389/fmed.2026.1999533

**Published:** 2026-10-08

**Authors:** Jingchun Song, Junjie Zeng, Xiaomin Song

**Affiliations:** 1 Department of Critical Care Medicine, The 908th Hospital of Joint Logistic Support Force, Nanchang, China; 2 Nanchang Key Laboratory of Thrombosis and Haemostasis, Nanchang, China

**Keywords:** COVID-19, disseminated intravascular coagulation, haemorrhagic DIC, hyperfibrinolysis, thrombotic DIC

## Abstract

**Background:**

COVID-19-associated coagulopathy has been predominantly characterised as a hypercoagulable state with fibrinolytic shutdown. However, the full phenotypic spectrum of disseminated intravascular coagulation (DIC) in COVID-19 remains incompletely characterised. This report adds two cases demonstrating that COVID-19-associated DIC can manifest as two distinct phenotypes at opposite ends of the coagulation spectrum.

**Case summary:**

We report two cases of severe COVID-19 with diametrically opposed DIC phenotypes. Case 1: a 23-year-old immunosuppressed male with SARS-CoV-2 bloodstream and central nervous system infection developed haemorrhagic DIC, characterised by severe hypofibrinogenemia (0.76 g/L), markedly elevated PIC (>40 μg/mL), and preserved platelet counts (>100 × 10^9^/L). He was successfully treated with titrated tranexamic acid. Case 2: a 54-year-old previously healthy female with severe COVID-19 pneumonia developed thrombotic DIC, characterised by significant thrombocytopenia (platelets 65 × 10^9^/L, nadir 40 × 10^9^/L), multi-organ injury, and lower extremity venous thrombosis. She was successfully treated with low-molecular-weight heparin.

**Conclusion:**

COVID-19-associated DIC spans two phenotypes—thrombotic (platelet consumption, requiring anticoagulation) and haemorrhagic (fibrinogen consumption with hyperfibrinolysis, requiring antifibrinolytics)—validating the 2025 ISTH classification and highlighting the need for phenotype-specific therapy guided by serial coagulation monitoring.

## 1 Introduction

Coronavirus Disease 2019 (COVID-19) -associated coagulopathy (CAC) is a distinctive systemic haemostatic disorder observed in patients with moderate to severe SARS-CoV-2 infection, characterised by endothelial dysfunction, dysregulated haemostasis, and a prothrombotic state (1). The incidence of thrombotic events in critically ill COVID-19 patients is substantial, with early evaluations reporting a 31% incidence of thrombotic complications among intensive care unit (ICU) patients, of which venous thromboembolic events accounted for approximately 27% (2). A meta-analysis of Venous Thromboembolism (VTE) in hospitalized COVID-19 patients further corroborated these findings, and depending on the investigations performed and thromboprophylactic regimens used, the reported incidence of VTE varies between 6% and 86%, with most trials reporting prevalences between 15% and 40%—significantly higher than the 5%–30% prevalence described for non-COVID-related critical illness (3). Disseminated intravascular coagulation (DIC) occurs in approximately 6.2% of COVID-19 patients overall, and up to 71.4% of non-survivors meet International Society on Thrombosis and Haemostasis (ISTH) criteria for overt DIC (4).

Hypercoagulability with fibrinolytic shutdown represents the most prominent coagulopathy phenotype in CAC. Patients universally exhibit elevated D-dimer, fibrinogen, and fibrin degradation products, with significantly higher levels observed in severe cases (5). Mechanistically, COVID-19-associated inflammation upregulates plasminogen activator inhibitor-1 (PAI-1) — the most potent physiological inhibitor of fibrinolysis—resulting in a state of fibrinolytic resistance (6). Elevated PAI-1 impairs plasmin generation, reducing fibrin degradation and promoting microvascular fibrin deposition and thrombosis. Beyond hypercoagulability and thromboembolic events, CAC can also manifest as thrombocytopenia, coagulation factor depletion, and, in severe cases, progression to overt DIC.

Clinically, CAC encompasses a broad spectrum ranging from microvascular thrombosis to overt DIC—the latter representing the decompensated, life-threatening stage of systemic coagulation activation. Venous thromboembolism, including pulmonary embolism and lower extremity deep vein thrombosis, is among the most common manifestations of CAC, with significantly increased incidence in critically ill patients (7). Microvascular thrombosis presents as multi-organ injury—acute kidney injury, myocardial injury, stroke, and acral ischaemia (e.g., “COVID toes”) (8, 9). As the disease advances, widespread thrombosis and coagulation factor consumption may culminate in consumptive coagulopathy, i.e., overt DIC.

Importantly, while the majority of CAC patients present with a hypercoagulable, hypofibrinolytic phenotype, a small number of case reports have recently described a hyperfibrinolytic haemorrhagic DIC phenotype in COVID-19 (10). This phenotypic heterogeneity suggests that CAC is not a single entity but rather a spectrum, and that overt DIC in COVID-19 can manifest in two distinct forms—thrombotic or haemorrhagic. In 2025, the International Society on Thrombosis and Haemostasis (ISTH) released updated definitions and diagnostic criteria for DIC, formally classifying overt DIC into thrombotic and haemorrhagic phenotypes (11). Against this backdrop, we report two cases of COVID-19-associated overt DIC manifesting as thrombotic DIC and haemorrhagic DIC, respectively, to illustrate the phenotypic diversity of DIC in COVID-19 and its implications for clinical management.

## 2 Case Description

### 2.1 Case 1: haemorrhagic DIC

A 23-year-old male with a one-year history of nephrotic syndrome was initially admitted to the nephrology department due to an upper respiratory tract infection. He had no prior history of blood disorders, thrombosis, or bleeding diathesis. He had been maintained on chronic immunosuppressive therapy, including tapered prednisone, tacrolimus, and rituximab administered three months prior. Chronic corticosteroid use had resulted in moon facies, abdominal distension, extensive purple atrophic striae, and femoral head necrosis (Figure 1A). During his hospital stay, he developed fever, headache, vomiting, and diarrhoea. Pre-transfer imaging studies revealed no definite intracranial abnormalities on head CT or brain MRI (plain, contrast-enhanced, and diffusion-weighted sequences); bilateral ethmoid sinusitis was noted on head CT, and minor dependent opacities were observed in both lower lobes on chest CT (Figure 1B). Lower extremity venous ultrasound showed no evidence of thrombosis. Despite these unremarkable imaging findings, his clinical condition deteriorated, prompting transfer to the intensive care unit (ICU). On ICU admission, vital signs were stable; physical examination revealed mild bilateral lower extremity oedema and negative meningeal signs.

**Figure 1:**
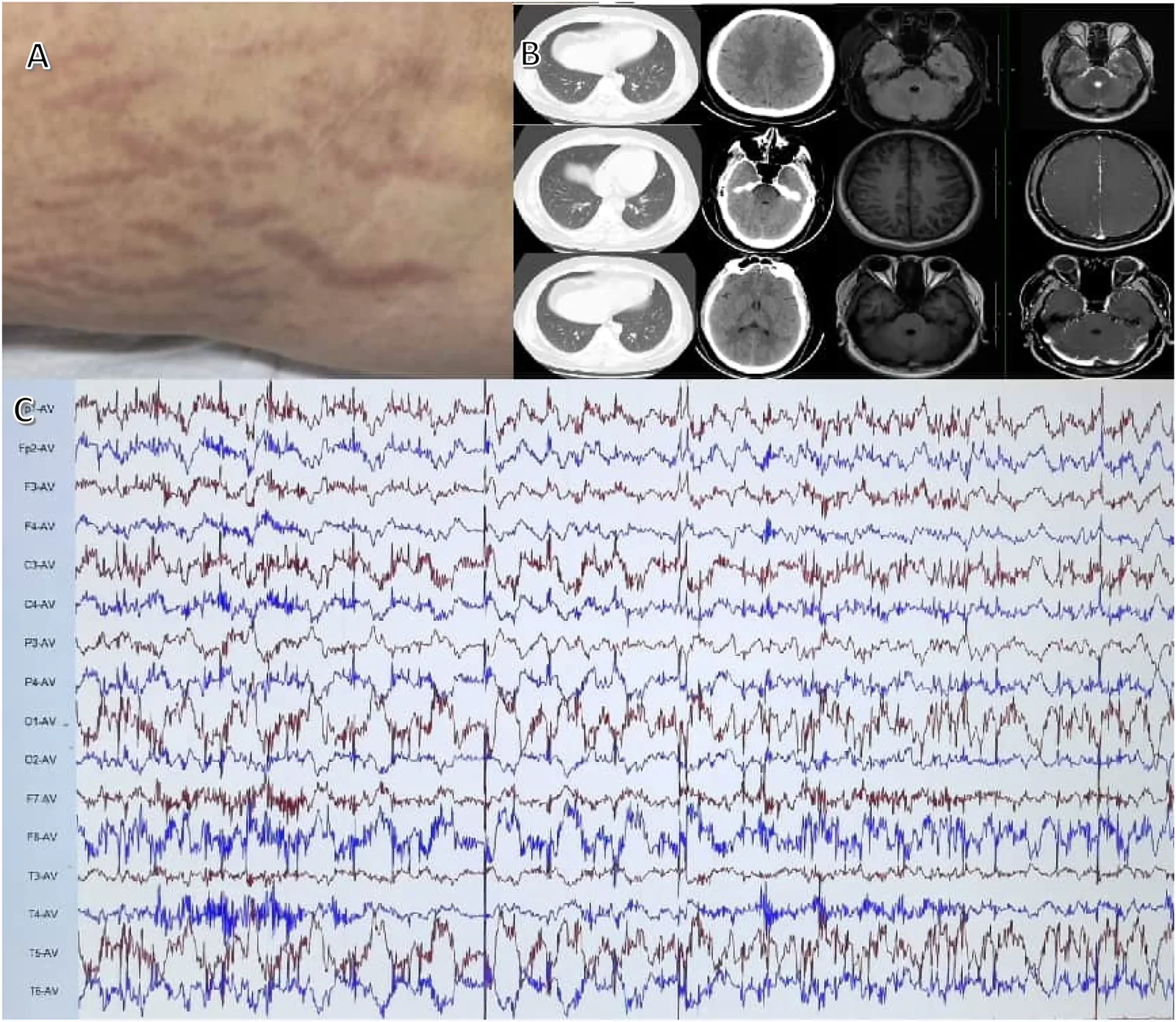
Clinical and diagnostic findings in the patient with haemorrhagic COVID-19 associated DIC (case 1). **(A)** Abdominal wall showing extensive purple atrophic striae, a characteristic cutaneous manifestation of chronic corticosteroid therapy. **(B)** Chest CT, head CT, and brain MRI revealing no definite parenchymal abnormalities. **(C)** Electroencephalogram demonstrating generalised epileptiform discharges, consistent with status epilepticus, which developed on day 3 of ICU admission.

Laboratory findings on admission (Table 1) included: prothrombin time (PT) 17.6s, activated partial thromboplastin time (APTT) 56.7s, fibrinogen 0.7 g/L, D-dimer 460.18 mg/L, fibrin degradation products (FDP) 1,301 μg/mL, thrombomodulin (TM) 101.56 TU/mL (normal 3.82–13.35), thrombin-antithrombin complex (TAT) 571 ng/mL (normal 0–4.07), plasmin-α2-plasmin inhibitor complex (PIC) 57 μg/mL (normal 0–0.85), and tissue plasminogen activator-inhibitor complex (t-PAIC) 42.77 ng/mL. Platelet count was 258 × 10^9^/L and remained >100 × 10^9^/L throughout. Using the 2025 ISTH updated criteria, the overt DIC score was 5: platelet count 258 × 10^9^/L (0 points), PT prolongation of 3 s (1 point), D-dimer 312 mg/L (>7× upper limit of normal, ULN, 3 points), and fibrinogen 0.76 g/L (1 point). Markedly elevated PIC (>60× ULN) indicated excessive plasmin generation; elevated TAT (>140× ULN) indicated concurrent thrombin generation. This profile confirmed haemorrhagic DIC (hyperfibrinolytic phenotype) according to the 2025 ISTH classification (11)—representing the decompensated, haemorrhagic end of the CAC spectrum. The patient received titrated continuous infusion of tranexamic acid (1 g loading dose followed by 125 mg/h, 4 days), combined with fibrinogen concentrate (7 g/day, 2 days) and prothrombin complex concentrate (2,400 IU). Following fibrinogen correction, lumbar puncture revealed an opening pressure of 200 mmH_2_O. Both blood and cerebrospinal fluid next-generation sequencing (NGS) identified SARS-CoV-2, confirming central nervous system infection. Nirmatrelvir/ritonavir was administered for its ability to penetrate the blood-brain barrier. On day 3, serum creatinine rose to 459.5 mmol/L with oliguria, prompting continuous renal replacement therapy (CRRT). The patient developed generalised seizures; electroencephalogram (EEG) revealed widespread epileptiform discharges (Figure 1C). Emergent management included intubation, anticonvulsants, and intracranial pressure control. Nafamostat mesylate was used for regional anticoagulation during CRRT.

**Table 1:** Admission coagulation profiles of both cases.

Parameter	Case 1 (Haemorrhagic DIC)	Case 2 (Thrombotic DIC)	Reference range
Platelets (×10^9^/L)	258 (consistently >100)	76 (at onset)/65 (ICU)	125–350
PT (s)	17.6	17.10 (at onset)/11.9 (ICU)	10–14
INR	1.61	1.56 (at onset)/0.96 (ICU)	0.8–1.3
APTT (s)	56.7	79.50 (at onset)/32.7 (ICU)	22–35
Fibrinogen (g/L)	0.7	1.6 (at onset)/1.48 (ICU)	2.0–4.0
D-dimer (mg/L)	460.18	20.7 (at onset)/5.10 (ICU)	≤0.5
FDP (μg/mL)	1,301	11.59	≤5.0
Thrombomodulin (TU/mL)	101.56	27.22	3.82–13.35
TAT (ng/mL)	571	7.61	≤4.0
PIC (μg/mL)	57	1.32	≤0.8
t-PAIC (ng/mL)	42.77	15.66	≤17.13
AT (%)	78	56	80–120

PT, prothrombin time; INR, international normalized ratio; APTT, activated partial thromboplastin time; FDP, fibrin degradation products; TAT, thrombin-antithrombin complex; PIC, plasmin-α2-plasmin inhibitor complex; t-PAIC, tissue plasminogen activator-inhibitor complex; AT, antithrombin; ICU, intensive care unit.

Post-treatment coagulation parameters normalised progressively (Table 2): FDP decreased from 1,301 to 62 μg/mL; D-dimer from 468.18 to 29.43 mg/L; fibrinogen rose from 0.7 to 3.4 g/L by day 4. No further seizures occurred. The patient was extubated on day 11 and transferred to nephrology on day 15. At one-month follow-up, he remained well without neurological sequelae.

**Table 2:** Dynamic changes in coagulation parameters during treatment (case 1: haemorrhagic DIC).

Parameter	Day 0	Day 1	Day 2	Day 3	Day 4	Reference
Platelets (×10^9^/L)	258	273	276	340	237	125–350
PT (s)	16.4	17.6	15.3	13.7	13.3	10–14
APTT (s)	53.2	56.7	48.4	43.6	38.5	22–35
Fibrinogen (g/L)	2.0	0.7	1.6	2.0	3.4	2.0–4.0
D-dimer (mg/L)	73.72	460.18	140.12	95.47	29.43	≤0.5
FDP (μg/mL)	-	1,301	401	279	62	≤5.0
AT(%)	-	78	63	60	49	80–120
TAT (ng/mL)	-	571	205	78.4	26.6	≤4.0
PIC (μg/mL)	-	57	16	8.8	5.6	≤0.8
t-PAIC (ng/mL)	-	42.77	34.22	23.84	17.9	≤17.13
TM (TU/mL)	-	101.56	105.38	95.94	84.28	3.8–13.3
Therapeutic Interventions	–	TXA + FIB + PCC	TXA + FIB	TXA	-	–

PT, prothrombin time; APTT, activated partial thromboplastin time; FDP, fibrin degradation products; AT, antithrombin; TAT, thrombin-antithrombin complex; PIC, plasmin-α2-plasmin inhibitor complex; t-PAIC, tissue plasminogen activator-inhibitor complex; TM, thrombomodulin; TXA, tranexamic acid (continuous intravenous infusion); FIB, human fibrinogen concentrate (intermittent supplementation); PCC, prothrombin complex concentrate (intermittent supplementation).

### 2.2 Case 2: thrombotic DIC

A 54-year-old previously healthy female with no prior history of blood disorders, thrombosis, or bleeding diathesis presented on Day 0 with fever up to 40.0°C, accompanied by chest tightness and headache. Local symptomatic treatment achieved only partial improvement. On Day 2, she developed extensive ecchymoses involving the face and extremities (Figures 2A,B). On Day 4, she progressed to tachycardia and hypotension. Laboratory studies at the local hospital revealed: platelets 76 × 10^9^/L, PT 17.10s, APTT 79.50s, fibrinogen 1.6 g/L, D-dimer 20.7 mg/L, and creatinine 434.0 μmol/L; throat swab SARS-CoV-2 nucleic acid test was positive. Despite administration of fresh frozen plasma and anti-infective therapy, her clinical condition did not improve. She was transferred to our ICU on Day 5 with severe COVID-19 pneumonia, extensive ecchymoses, multi-organ injury, and consumptive coagulopathy—a clinical picture consistent with progression of COVID-19-associated coagulopathy (CAC) to the decompensated, thrombotic end of the DIC spectrum.

**Figure 2:**
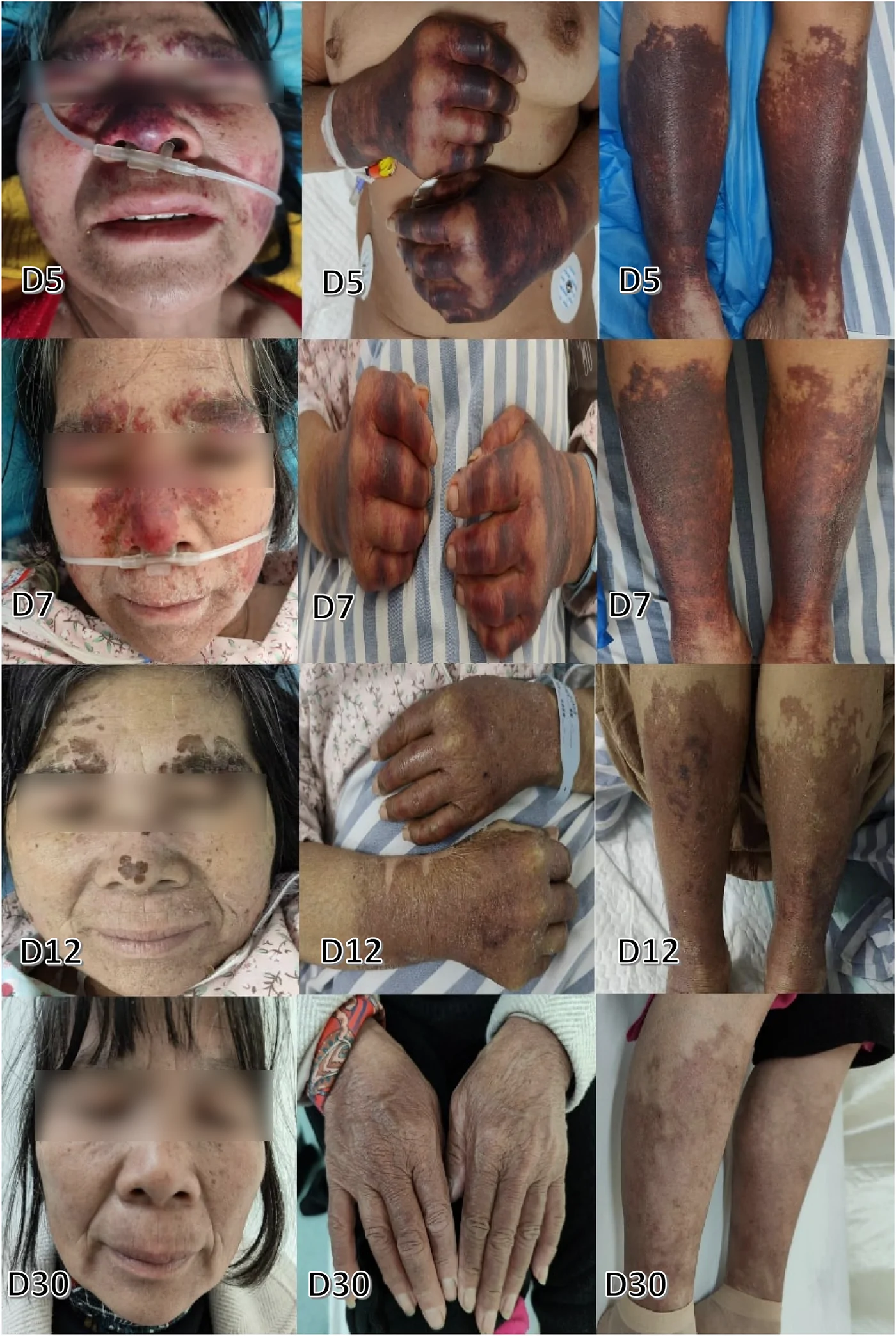
Progressive resolution of extensive purpura in the patient with COVID-19 associated thrombotic DIC (case 2, from day 5 to day 30).

On ICU admission (Table 1), she presented with septic shock (procalcitonin >50 ng/mL, requiring vasopressor support), acute respiratory distress syndrome (PaO_2_/FiO_2_ 145 mmHg), acute kidney injury (creatinine 292.8 μmol/L), and hepatic injury (alanine aminotransferase 101.7 U/L). Coagulation studies revealed: platelets 65 × 10^9^/L (nadir 40 × 10^9^/L on Day 7), PT 11.9s, APTT 32.7s, fibrinogen 1.48 g/L, D-dimer 5.10 μg/mL, FDP 11.59 μg/mL, antithrombin 56%, TM 27.22 TU/mL (normal 3.82–13.35), TAT 7.61 ng/mL, PIC 1.32 μg/mL, and t-PAIC 15.66 ng/mL. Lower extremity ultrasound revealed bilateral calf vein and posterior tibial vein thrombosis (deep vein thrombosis, DVT) (Figure 2C). Autoimmune workup was negative.

Diagnosis: Using the 2025 ISTH updated criteria, the overt DIC score based on Day 4 laboratory data was 5: platelets 76 × 10^9^/L (1 point), PT prolongation 17.10s (1 point), D-dimer 20.7 mg/L (>7× ULN, 3 points), and fibrinogen 1.6 g/L (0 points), consistent with overt DIC (≥5 points). On Day 5, the score decreased to 3 (platelets 1 point; D-dimer 5.10 μg/mL, >3× ULN but <7× ULN, 2 points; PT and fibrinogen 0 points), reflecting partial correction following initial interventions. The patient was diagnosed with thrombotic DIC (11), characterised by microvascular thrombosis (skin purpura/ecchymoses), macrovascular thrombosis (lower extremity DVT), multi-organ injury, and consumptive coagulopathy—representing the thrombotic end of the overt DIC spectrum arising from CAC. Unlike Case 1, fibrinolytic markers (PIC 1.32 μg/mL) were not elevated; instead, significant thrombocytopenia was the dominant feature, consistent with the thrombotic phenotype. The markedly elevated TM (27.22 TU/mL) indicated severe endothelial injury—the common upstream mechanism driving progression from CAC to overt DIC.

Treatment and outcome: The patient received low-molecular-weight heparin (nadroparin calcium) anticoagulation, methylprednisolone for anti-inflammatory effect, piperacillin-tazobactam for anti-infection, metoprolol tartrate for rate control, and supportive care including high-flow nasal oxygen, vasopressors, and intravenous fluid resuscitation. Purpura began resolving on Day 7 (Figures 2D,E). Platelet count recovered from its nadir of 40 × 10^9^/L on Day 7 to normal by Day 12. Repeat ultrasound confirmed resolution of venous thrombosis by Day 12. She was discharged cured without sequelae.

## 3 Discussion

These two cases provide a compelling illustration that COVID-19-associated DIC is not a monolithic entity but rather a spectrum of phenotypes, ranging from thrombotic DIC to haemorrhagic DIC, with SARS-CoV-2-induced endothelial injury as the common pathogenic driver. This phenotypic dichotomy aligns precisely with the 2025 ISTH updated classification, which now formally categorises DIC into thrombotic and haemorrhagic phenotypes (11).

### 3.1 Endothelial injury: the common upstream mechanism

SARS-CoV-2 directly infects endothelial cells via angiotensin-converting enzyme 2 (ACE2) receptor binding, triggering endothelial activation, dysfunction, and injury (1, 7). Post-mortem analyses of pulmonary vessels from patients with COVID-19 revealed severe endothelial injury associated with the presence of intracellular virus and disrupted cell membranes, with alveolar capillary microthrombi being nine times more prevalent than in patients with influenza (12). This endothelopathy is reflected by markedly elevated thrombomodulin in both of our cases (101.56 TU/mL in Case 1, 27.22 TU/mL in Case 2; normal 3.82–13.35), a sensitive biomarker of endothelial damage (13, 14). SARS-CoV-2 spike protein induces lung endothelial cell dysfunction and thrombo-inflammation, with S1 injection in ACE2-transgenic mice inducing early loss of lung endothelial thromboresistance as revealed by thrombomodulin loss and von Willebrand factor (VWF) increase (1, 15).

Endothelial injury triggers a cascade of inflammatory and procoagulant responses, including complement activation, platelet activation, and tissue factor upregulation (1, 7). Neutrophil extracellular traps (NETs) induce immunothrombosis at the site of infection through histone- and DNA-mediated thrombin generation, insufficient anticoagulation control, and inhibition of fibrinolysis (16). Elevated NETs in the blood of patients with COVID-19 correlate with thrombin generation, severity of respiratory status and risk of death. When infection is sufficiently severe, immunothrombosis disseminates into the systemic circulation, and DIC—associated with endothelial injury—ensues (7, 15). This aligns with the 2025 ISTH definition of DIC as “an acquired, life-threatening intravascular disorder characterised by systemic coagulation activation, dysregulated fibrinolysis, and endothelial injury, resulting in microthrombosis” (11).

### 3.2 Divergent phenotypes: role of host factors and disease dynamics

Why did these two patients develop diametrically opposed phenotypes? We propose several determinants (Table 3).

**Table 3:** Comparison of phenotypes: haemorrhagic DIC vs. Thrombotic DIC.

Feature	Case 1 (Haemorrhagic DIC)	Case 2 (Thrombotic DIC)
Age/sex	23-year-old male	54-year-old female
Underlying condition	Immunosuppressed (nephrotic syndrome)	Previously healthy
SARS-CoV-2 involvement	Bloodstream + CNS infection	Pulmonary (pneumonia)
2025 ISTH phenotype	Haemorrhagic DIC	Thrombotic DIC
ISTH DIC score (at onset)	5 (overt DIC)	5 (overt DIC)
Platelet trend	Consistently >100 × 10^9^/L	Dropped to 65 × 10^9^/L (nadir 40)
PIC	57 μg/mL (markedly elevated)	1.32 μg/mL (normal)
Fibrinogen	0.7 g/L (severely decreased)	1.6 g/L (mildly decreased)
D-dimer	460.18 mg/L (markedly elevated)	20.7 mg/L (elevated)
TM	101.56 TU/mL (markedly elevated)	27.22 TU/mL (elevated)
Primary pathology	Excessive fibrinolysis (haemorrhagic)	Microvascular + macrovascular thrombosis
Clinical manifestation	Bleeding risk, fibrinogen consumption	Purpura/ecchymoses, multi-organ injury, DVT
Key therapy	Tranexamic acid (antifibrinolytic)	LMWH (anticoagulation)
Outcome	Full recovery	Full recovery

#### 3.2.1 Immune status

Case 1 was profoundly immunosuppressed (prednisone, tacrolimus, rituximab), which may have attenuated the typical cytokine storm, resulting in relatively lower PAI-1 induction and allowing unchecked fibrinolysis. SARS-CoV-2 infection typically results in upregulation of PAI-1 and neuroserpin in the lungs, with an increase in fibrinolysis inhibitors associated with disease severity (1, 6). In the immunosuppressed host, this upregulation may be blunted, permitting a hyperfibrinolytic state to emerge. Case 2 was immunocompetent, mounting a full inflammatory response that drove extensive microvascular thrombosis (1, 7).

#### 3.2.2 Inflammatory response pattern

Classical CAC features fibrinolytic shutdown driven by elevated PAI-1 (6, 17). In critically ill patients with COVID-19, widespread inflammatory response and endothelial injury lead to systemic coagulation activation and depletion of coagulation factors and platelets (1, 7). In Case 1, the blunted inflammatory response may have failed to upregulate PAI-1 sufficiently, permitting hyperfibrinolysis to dominate. In Case 2, robust inflammation drove PAI-1 elevation, promoting microvascular thrombosis and extensive purpura/ecchymoses.

#### 3.2.3 Disease stage and viral burden

COVID-19 may evolve over time. In the early stage of SARS-CoV-2 infection, the fibrinolytic system is mostly inhibited, responsible for the development of hypofibrinolysis, promoting DIC, hyaline membrane formation, and pulmonary oedema (4, 5). In the advanced stage, a large amount of fibrin degradation products—including D-dimer—becomes the characteristic feature of hyperfibrinolysis (10). Case 1 presented with advanced multisystem involvement (bloodstream infection, CNS, kidneys), suggesting later-stage disease with exhaustion of fibrinolytic inhibitors (4). Case 2 presented with severe pulmonary involvement and early coagulopathy, consistent with the classical hypofibrinolytic phenotype (5).

#### 3.2.4 Dynamic evolution of coagulation parameters and platelet trends

The temporal changes observed in Case 2—from an ISTH score of 5 at onset (platelets 76, PT 17.10s, D-dimer 20.7 mg/L) to 3 at ICU admission (platelets 65, PT 11.9s, D-dimer 5.10 μg/mL)—illustrate the dynamic nature of CAC (4, 18). The partial normalisation of PT and D-dimer following initial interventions, despite persistent and even worsening thrombocytopenia, highlights that coagulation parameters in COVID-19 are not static but evolve with disease progression and therapeutic interventions (1, 4). This dynamic evolution further complicates phenotypic classification and underscores the need for serial monitoring to guide therapy (11). Crucially, Case 1 maintained platelet counts consistently >100 × 10^9^/L throughout the entire clinical course, while Case 2's platelet count dropped to 65 × 10^9^/L (nadir 40 × 10^9^/L)—a distinguishing feature that reflects the fundamental pathophysiological difference between haemorrhagic DIC (platelet-sparing, fibrinogen-consuming) and thrombotic DIC (platelet-consuming, microthrombotic) phenotypes (11).

#### 3.2.5 Site of infection

The two cases differed markedly in the primary site of SARS-CoV-2 infection: Case 1 had central nervous system infection (confirmed by CSF NGS), while Case 2 had pulmonary infection (severe COVID-19 pneum19onia). This difference in infection site may have contributed to the divergent coagulation phenotypes through organ-specific inflammatory responses and differential endothelial activation patterns (7).

#### 3.2.6 Host genetic factors

Individual variations in fibrinolytic pathway components—including polymorphisms in PAI-1, tissue plasminogen activator, or fibrinogen genes—may predispose certain individuals to divergent phenotypes (6).

### 3.3 Thrombotic DIC in COVID-19

Case 2 exemplifies the thrombotic DIC phenotype according to the 2025 ISTH classification (11), characterised by microvascular thrombosis, multi-organ dysfunction, and consumptive thrombocytopenia. This phenotype represents the progression of COVID-19-associated coagulopathy from a compensated (non-overt) state to overt DIC, with widespread microvascular thrombotic burden becoming the dominant pathology.

In thrombotic DIC, widespread microvascular thrombosis leads to platelet consumption and thrombocytopenia (7, 11). The extensive purpura/ecchymoses observed in Case 2 represent cutaneous manifestations of microvascular thrombosis, reflecting platelet aggregation and consumption in the dermal microvasculature (7). Beyond the skin, microvascular thrombosis extends to multiple organ systems—acute kidney injury (8), acute respiratory distress syndrome (5), and hepatic injury in this case—resulting from ischaemic damage due to microvascular occlusion. Concurrent macrovascular thrombosis, as demonstrated by DVT in Case 2, may also coexist (13). The combination of microvascular and macrovascular thrombosis represents the full spectrum of thrombotic complications in this phenotype (13, 19).

The markedly elevated TM (27.22 TU/mL; normal 3.82–13.35) in Case 2 provides direct evidence of severe endothelial injury. This endothelopathy, combined with complement activation and NETosis, creates a prothrombotic milieu that drives systemic microvascular thrombosis (17, 20). In thrombotic DIC, laboratory findings are dominated by progressive thrombocytopenia due to platelet consumption, with relatively preserved fibrinogen levels—distinguishing this phenotype from haemorrhagic DIC, where fibrinogen consumption predominates with platelet preservation (21, 22).

Importantly, thrombotic microangiopathy (TMA) is a distinct clinical entity that requires differential diagnosis from DIC (23). TMA is characterised by mechanical haemolytic anaemia (schistocytes on peripheral blood smear), severe thrombocytopenia, and organ injury, typically resulting from ADAMTS13 (a disintegrin and metalloproteinase with a thrombospondin type 1 motif, member 13) deficiency or complement dysregulation (23). In contrast, DIC is driven by systemic coagulation activation and does not typically feature schistocytes or severe ADAMTS13 deficiency (11). Our Case 2 had no schistocytes on peripheral blood smear and negative autoimmune workup (ADAMTS13 activity was not measured, but the clinical picture was more consistent with DIC than with primary TMA). The absence of schistocytes, together with the presence of prolonged PT and elevated D-dimer out of proportion to isolated TMA, supported the diagnosis of thrombotic DIC rather than TMA (11, 18).

The patient's full recovery without skin necrosis, amputation, or other sequelae underscores the importance of early recognition and prompt anticoagulation (13, 24). Early anticoagulation with low-molecular-weight heparin in thrombotic DIC aims to halt the progression of microvascular thrombosis, preserve organ function, and prevent consumptive thrombocytopenia from progressing to life-threatening levels (13, 25).

### 3.4 Hyperfibrinolytic DIC in COVID-19

Hyperfibrinolytic DIC in COVID-19 has been rarely reported (10). COVID-19-associated hyperfibrinolysis has been described, with elevated D-dimer levels attributed to both hyperfibrinolysis and hypercoagulation (26, 27). Plasmin is a key player in fibrinolysis and is involved in the cleavage of many virus envelope proteins, including SARS-CoV.

However, detailed characterisation of hyperfibrinolytic DIC with comprehensive thrombus molecular markers (TAT, PIC, t-PAIC) has been lacking (28). To confirm the diagnosis of enhanced-fibrinolytic-type DIC, measurement of not only the coagulation activation marker TAT, but also the fibrinolysis activation marker PIC is essential. Appropriate assessment of both coagulation and fibrinolysis on a case-by-case basis may lead to improved outcomes for COVID-19. Our Case 1 may be among the first to document COVID-19-associated DIC with such pronounced hyperfibrinolysis confirmed by comprehensive thrombus molecular markers. The markedly elevated PIC (>40 μg/mL) directly reflects excessive plasmin generation; elevated TAT (>120 ng/mL) confirms concurrent thrombin generation; and severe hypofibrinogenemia (0.76 g/L) reflects consumption. This constellation of findings confirms a true hyperfibrinolytic DIC phenotype rather than simply elevated D-dimer from other causes (10). Importantly, platelet count remained consistently >100 × 10^9^/L throughout the course—a hallmark of the haemorrhagic DIC phenotype according to the 2025 ISTH classification, reflecting a platelet-sparing consumption pattern (11).

The use of tranexamic acid in this patient proved beneficial, with progressive normalisation of coagulation parameters. Tranexamic acid modulates coagulopathy through the suppression of fibrinolysis and, hence, could prevent DIC during SARS-CoV-2 infection (10). In critical COVID-19 patients, hypercoagulation and hyperfibrinolysis responses with elevated levels of FDPs have been reported (10). The use of tranexamic acid may be indicated in DIC with enhanced fibrinolysis and severe haemorrhage (29). However, recovery from DIC is dependent on endogenous fibrinolysis to break down disseminated thrombi—a process inhibited by tranexamic acid. Therefore, tranexamic acid must be used with extreme caution and only in confirmed hyperfibrinolytic DIC.

### 3.5 Diagnostic considerations: 2025 ISTH DIC criteria and phenotypic classification

A critical conceptual distinction must be made between CAC as a broad spectrum and overt DIC as its decompensated stage. To help readers visualise this hierarchy, we provide a conceptual framework in Figure 3, which illustrates the continuum from SARS-CoV-2 infection through endothelial injury to CAC, and its progression to the two overt DIC phenotypes.

**Figure 3:**
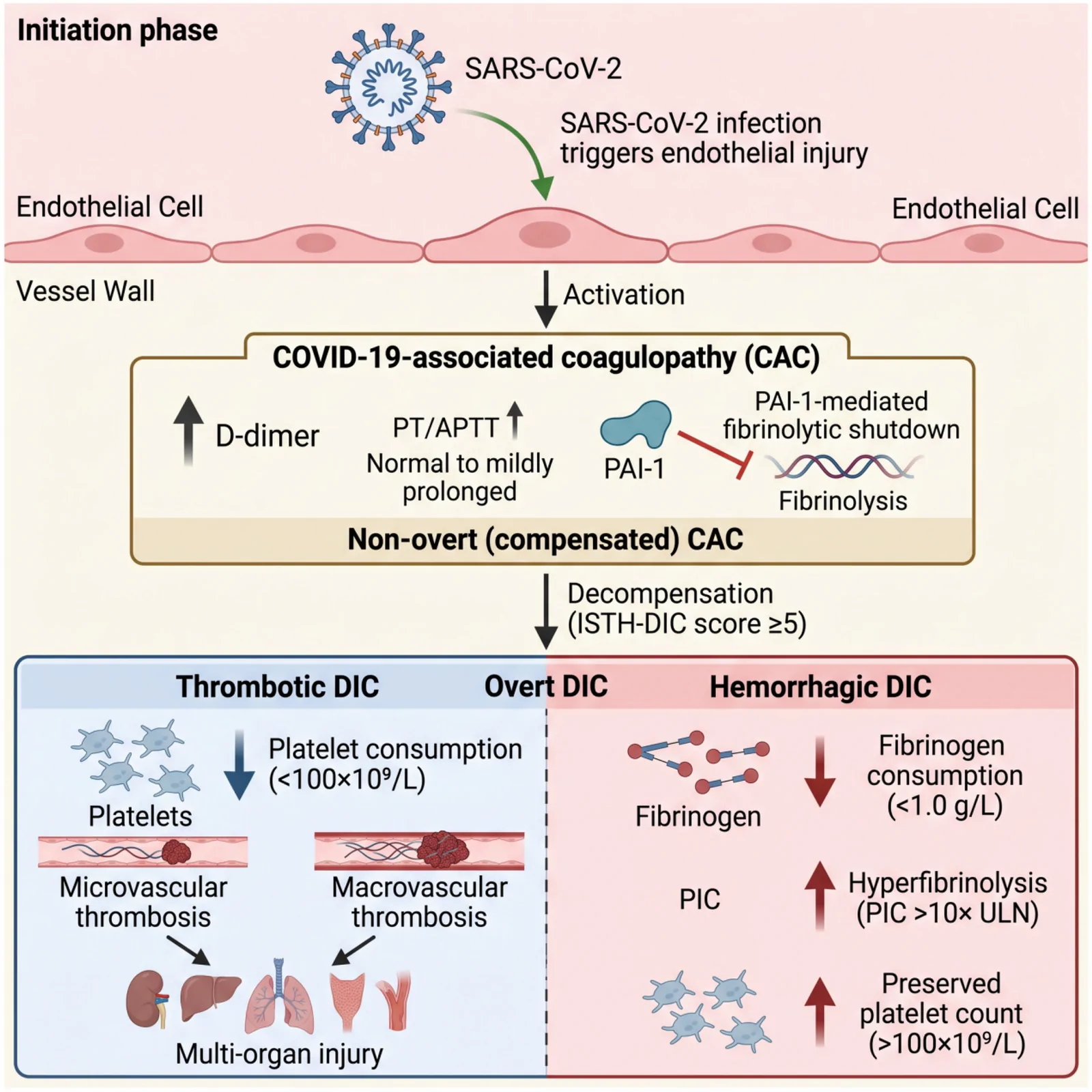
Conceptual framework of CAC and overt DIC. CAC, COVID-19-associated coagulopathy; DIC, disseminated intravascular coagulation; PAI-1, plasminogen activator inhibitor-1; PIC, plasmin-α2-plasmin inhibitor complex; ULN, upper limit of normal.

The ISTH overt DIC scoring system was updated in 2025 with revised D-dimer thresholds: levels >3× and >7× the upper normal limit now correspond to 2 and 3 points, respectively (11). A cumulative score of ≥5 is considered diagnostic of overt DIC (11, 26). A major innovation in the 2025 update is the phase-based classification of DIC: Pre-DIC, early-phase DIC, and overt DIC (11). Early-phase DIC—also referred to as subclinical or compensated DIC—is characterised by laboratory abnormalities preceding clinical symptoms. Overt DIC represents the advanced stage with clear evidence of coagulopathy and organ failure. Importantly, the criteria also classify overt DIC into thrombotic and haemorrhagic phenotypes (30):

Thrombotic DIC (as in Case 2): characterised by significant thrombocytopenia (platelets <100 × 10^9^/L), multi-organ dysfunction, and relatively preserved fibrinogen. The scoring profile is dominated by platelet count and D-dimer elevation.

Haemorrhagic DIC (as in Case 1): characterised by severe hypofibrinogenemia (fibrinogen <1.0 g/L), markedly elevated PIC (>10× ULN), preserved platelet counts (>100 × 10^9^/L), and bleeding risk (11, 28). The scoring profile is dominated by D-dimer, PT prolongation, and low fibrinogen.

Interestingly, both of our overt DIC cases met the diagnostic threshold for overt DIC at their respective time points, but via entirely different scoring profiles (Table 4). Case 1 (haemorrhagic DIC) scored predominantly on D-dimer (3 points), PT prolongation (1 point), and fibrinogen (1 point)—reflecting the haemorrhagic/hyperfibrinolytic phenotype, with platelets consistently >100 × 10^9^/L (0 points). Case 2 (thrombotic DIC) at onset scored on platelet count (1 point), PT (1 point), and D-dimer (3 points)—reflecting the thrombotic phenotype with consumptive coagulopathy. The subsequent evolution of Case 2's score from 5 to 3 at ICU admission demonstrates that a single snapshot may not capture the full picture and that serial monitoring is essential (15, 26).

**Table 4:** ISTH overt DIC scoring comparison between the two cases (2025 updated criteria).

Parameter	Score	Case 1 (at ICU) haemorrhagic phenotypes	Case 2 (at onset) thrombotic phenotypes
Platelet count (×10^9^/L)	>100 = 0; 50–100 = 1; <50 = 2	258 → 0	76 → 1
PT prolongation (s)	<3 = 0; ≥3–<6 = 1; ≥6 = 2	PT 17.6s → 1	PT 17.10s → 1
D-dimer(mg/L)	≤3× ULN = 0; >3× ULN = 2; >7× ULN = 3	460.18 (>7× ULN) → 3	20.7 (>7× ULN) → 3
Fibrinogen (g/L)	>1.0 = 0; <1.0 = 1	0.7 → 1	1.6 → 0
Total score	≥5 = overt DIC	5	5

The 2025 ISTH updated criteria classify DIC into thrombotic and haemorrhagic phenotypes. Case 1 (haemorrhagic DIC) scored predominantly on D-dimer (3), PT (1), and fibrinogen (1)—reflecting consumption and hyperfibrinolysis, with platelets consistently >100 × 10^9^/L. Case 2 at onset (thrombotic DIC) scored on platelets (1), PT (1), and D-dimer (3)—reflecting microvascular/macrovascular thrombosis with consumptive thrombocytopenia.

Furthermore, the ISTH criteria may not be optimal for early recognition of non-overt COVID-19 coagulopathy. The COVID-19 coagulopathy diagnostic criteria was proposed (27), which include: (1) platelet count <150 × 10^9^/L; (2) D-dimer >2× upper normal limit; (3) PT prolongation >1 s or INR >1.2; and (4) presence of thrombosis (macro-thrombosis and/or micro-thrombosis) (9, 24). These criteria better capture the thrombotic phenotype of CAC in its compensated phase, as illustrated by the early presentation of Case 2.

### 3.6 Clinical implications: phenotype-specific therapy

Recognition of CAC phenotypic diversity has direct therapeutic implications. In classical CAC with fibrinolytic shutdown, anticoagulation is the mainstay (24, 31). In haemorrhagic DIC, antifibrinolytic therapy with tranexamic acid is indicated (10)—a treatment that would be contraindicated in classical CAC and thrombotic DIC due to thrombosis risk (31, 32). In thrombotic DIC, anticoagulation with LMWH is essential (13, 24). Misclassifying these phenotypes could be fatal: giving tranexamic acid to a patient with microvascular thrombosis would exacerbate clotting; giving anticoagulation alone to a patient with hyperfibrinolysis would not address the primary pathology (33). Table 5 summarises the therapeutic implications of phenotypic classification according to the 2025 ISTH framework (11).

**Table 5:** COVID-19-associated DIC phenotypes: from non-overt to overt DIC.

Phenotype	DIC stage	Fibrinolytic status	Platelet trend	Fibrinogen	Primary pathology	ISTH score	Primary therapy
Classical CAC	Non-overt DIC (Compensated)	Hypofibrinolysis (high PAI-1)	Normal to mildly decreased	Normal to elevated	Hypercoagulability, thrombosis risk	<5	Anticoagulation
Thrombotic DIC	Overt DIC	Microvascular/macrovascular thrombosis	Decreased (<100 × 10^9^/L)	Normal to mildly decreased	Platelet consumption, multi-organ injury	≥5	Anticoagulation + supportive therapy
Haemorrhagic DIC	Overt DIC	Hyperfibrinolysis (high PIC)	Consistently >100 × 10^9^/L	Severely decreased (<1.0 g/L)	Fibrinogen consumption, bleeding risk	≥5	Antifibrinolytics + replacement

CAC, COVID-19-associated coagulopathy; PAI-1, plasminogen activator inhibitor-1; PIC, plasmin-α2-plasmin inhibitor complex; ISTH, International Society on Thrombosis and Haemostasis.

### 3.7 Limitations

This report has several limitations inherent to a two-case series. The mechanisms underlying phenotypic divergence remain speculative and require further investigation. Comprehensive assessment of fibrinolytic regulators (e.g., PAI-1 antigen and activity, protein C and S levels) was not performed in all cases, which would have provided additional mechanistic insights (34, 35). The generalisability of these findings to other patient populations requires confirmation in larger cohorts.

## 4 Conclusions

In conclusion, COVID-19-associated DIC manifests as two distinct phenotypes—thrombotic DIC with platelet consumption and multi-organ injury, and haemorrhagic DIC with fibrinogen consumption and hyperfibrinolysis—each requiring opposite therapeutic strategies: anticoagulation vs. antifibrinolytics, respectively. These cases validate the 2025 ISTH phenotypic classification and demonstrate that the same endothelial injury can produce diametrically opposed coagulation phenotypes depending on host and disease factors.

## Data Availability

The original contributions presented in the study are included in the article/Supplementary Material, further inquiries can be directed to the corresponding author/s.
